# Comparative analysis of general anaesthesia with and without dexmedetomidine in emergence agitation

**DOI:** 10.6026/973206300214231

**Published:** 2025-11-15

**Authors:** Shalini Jain, Rasna K.P, Mohan Babu Nema, Manish Banjare

**Affiliations:** 1 Department of Anaesthesia, MGM Medical College, Indore, Madhya Pradesh, India

**Keywords:** Dexmedetomidine, emergence agitation, general anaesthesia, maxillofacial surgery, sedation, hemodynamic stability

## Abstract

Emergence agitation (EA) is a common concern in maxillofacial surgeries under general anaesthesia. Therefore, it is of interest to evaluate the role of intraoperative dexmedetomidine infusion in its prevention. Ninety ASA I-II adult patients were randomized into two groups general anaesthesia alone (Group A) and general anaesthesia with dexmedetomidine (Group B). Dexmedetomidine significantly reduced the incidence and severity of EA (p <l0.001) improved sedation scores and provided better hemodynamic stability without increasing pain, nausea or vomiting. Bradycardia was more frequent in the dexmedetomidine group but also manageable making it a safe and effective adjuvant for enhancing recovery quality.

## Background:

Emergence agitation (EA) is a post-anesthesia complication seen during early recovery, marked by confusion, restlessness, hyperactivity and disorientation. Though it can happen in all age groups, it is more frequent in young adults and elderly patients [1]. Incidence in adults is reported between 20% to 30% making it a relevant issue in postoperative care [2]. EA usually appears within 15-30 minutes of anesthesia emergence and is often short-lived, but if unmanaged, may cause self-extubation, accidental catheter removal, aspiration, hypoxia, trauma or even cardiac arrest [3, 4]. The exact pathophysiology of EA is still not clear. Proposed risk factors include intense pain, prolonged surgery, preoperative anxiety and irritating stimuli like endotracheal tubes or catheters. Surgeries involving face, breast or upper abdomen tend to show higher EA risk. In particular, ENT and maxillofacial surgeries especially nasal procedures have shown EA rates between 4.7% and 27.3% [5]. Sevoflurane is commonly used due to its fast induction and emergence, low blood-gas solubility and stable hemodynamics. Its rapid washout is linked with increased EA incidence [6]. Non-pharmacological methods like verbal reassurance or physical restraint show poor success. Pharmacological agents are more effective, but drugs like propofol, opioids or clonidine often delay recovery due to over-sedation [7, 8]. Dexmedetomidine a selective α2-agonist provides sedation, analgesia and sympatholysis without respiratory depression [9]. It reduces opioid use, stabilizes hemodynamics, prevents nausea and promotes smooth recovery [10]. Although dexmedetomidine is well studied in children for EA prevention, adult data remains limited. Therefore, it is of interest to report the effect of intraoperative dexmedetomidine infusion on emergence agitation in adults undergoing maxillofacial surgeries.

## Materials and Methods:

This cross-sectional observational analytical study was carried out in the Department of Anaesthesiology, M.G.M. Medical College and M.Y. Hospital, Indore, Madhya Pradesh after approval from the Institutional Ethics and Scientific Review Committee. Study duration was 12 months from October 2023- October 2024. Ninety patients scheduled for elective maxillofacial surgeries were included based on eligibility. Study objectives and procedures were explained in vernacular language and informed written consent was taken. Confidentiality was maintained. Sample size was calculated using G*Power software with support from a statistician to ensure adequate study power. Patients were randomized into two equal groups (n = 45 each) using computer-generated numbers:

[1] Group A: General anaesthesia alone

[2] Group B: General anaesthesia with intraoperative dexmedetomidine infusion

## Inclusion criteria: 

Adults aged 18-60 years, ASA grade I or II, either sex, posted for elective maxillofacial surgery under general anaesthesia.

## Exclusion criteria: 

Patients with BMI >35 kg/m^2^, allergy to study drugs, history of substance abuse, cognitive disorders, cardiovascular/systemic disease or on-going use of beta-blockers, α2 agonists or opioids.

## Anaesthesia protocol:

All patients underwent standard pre-anaesthesia workup. In OT, routine monitors were attached ECG, NIBP, SpO_2_ and EtCO_2_. IV access secured. Baseline vitals noted.

## Premedication: 

IV glycopyrrolate 0.2 mg and IV fentanyl 2 µg/kg. Induction with IV propofol (2-3 mg/kg) and IV succinylcholine (1.5-2 mg/kg). Intubation (oro/nasotracheal) done per surgical need.

## Maintenance:

50% O_2_ + 50% N_2_O with sevoflurane and IV atracurium as needed.

## Dexmedetomidine Protocol (Group B):

Patients received IV dexmedetomidine loading dose 1 µg/kg over 10 min (in 100 mL NS), followed by continuous infusion at 0.4 µg/kg/h, stopped at sevoflurane discontinuation (defined as TIME ZERO).

## Outcome Assessment:

Emergence agitation assessed using Riker Sedation-Agitation Scale (SAS). Recovery quality assess by 4-point cough scale, 4-point nausea-vomiting scale and pain using Numerical Rating Scale (NRS). Sedation evaluated by Ramsay Sedation Scale (RSS). Hemodynamics (HR, SBP, DBP, MAP, EtCO_2_, and SpO_2_) recorded at intraoperative intervals. If SAS >7, IV propofol 0.25 mg/kg given and event documented.

## Statistical analysis:

Data were entered in Excel, analysed with SPSS v25. Continuous data as mean ± SD, categorical as frequencies (%). Intergroup comparison had done use unpaired t-test (continuous) and Chi-square test (categorical). Fisher's exact test applied when expected frequency <5. A p-value <0.05 consider statistically significant. Results show in tables and graphs.

## Results:

A total of 90 patients were enrolled and equally divided into two groups Group A (General Anaesthesia alone) and Group B (General Anaesthesia with Dexmedetomidine infusion). Baseline demographic and clinical characteristics including age, height, weight, gender, ASA grade and duration of surgery were statistically comparable between both groups, confirming proper group matching before outcome assessment (p > 0.05 for all) (Table 1 - see PDF). Postoperative evaluation revealed a significantly lower incidence of emergence agitation in Group B. As per Riker Sedation-Agitation Scale, 64.4% of patients in Group B remained calm and cooperative (Grade 4), while none experienced severe agitation (Grades 6 or 7). In contrast, more than 50% of Group A patients had higher agitation grades (Grades 5-7). The difference was statistically significant (p < 0.001) (Table 2 - see PDF). No significant intergroup differences were observed in postoperative cough, nausea-vomiting scores, or pain levels. In both groups, over 75% had no cough or vomiting and about half of patients reported no postoperative pain (Table 2 - see PDF). Intraoperative hemodynamic monitoring showed that Group B had significantly lower heart rate, systolic, diastolic and mean arterial pressures during the mid-to-late phases of surgery (p < 0.05), reflecting the expected sedative and sympatholytic effect of dexmedetomidine. Group A maintained more consistent hemodynamics beyond 30 minutes. However the oxygen saturation (SpO_2_) was well-preserved and comparable across both groups throughout (p > 0.05), indicating maintained oxygenation in all patients. Sedation analysis using the Ramsay Sedation Scale revealed that Group B achieved superior sedation control, with 64.4% of patients in the optimal Grade 2 range, compared to only 28.9% in Group A. Furthermore, 53.3% of patients in Group A were agitated (Grade 1), while only 11.1% of Group B showed the same. The difference was statistically significant (p = 0.000) (Table 3 - see PDF). Regarding adverse effects, bradycardia was significantly higher in Group B (22.2%) compared to Group A (6.7%) (p = 0.036), confirming the known bradycardic tendency of dexmedetomidine. Hypotension was slightly more frequent in Group B (17.8%) than in Group A (6.7%), but this difference was not statistically significant (p = 0.108). No significant differences were noted for nausea-vomiting (p = 0.803) or requirement for intervention for emergence agitation (p = 0.459) (Table 4 - see PDF). Group B demonstrated significantly better outcomes in terms of emergence agitation control and sedation quality without increasing major adverse effects, except for a modest rise in bradycardia which was clinically manageable.

## Discussion:

Intraoperative dexmedetomidine infusion significantly reduced the incidence and severity of emergence agitation (EA) in adult maxillofacial surgery patients with 64.4% achieving calm, cooperative sedation (Riker Grade 4) versus 50% with higher agitation grades in controls (p < 0.001). These findings align with recent meta-analyses demonstrating dexmedetomidine's superior efficacy in preventing postoperative delirium across diverse surgical populations [11, 12]. Sevoflurane's rapid emergence kinetics attributed to its low blood-gas solubility is a well-recognized risk factor for EA in adults [13]. Recent network meta-analysis of nasal surgeries a maxillofacial*- specialty with comparable anatomical field complexity identified dexmedetomidine as the most effective pharmacological intervention (RR 0.27, 95% CI 0.20-0.36) compared to ketamine, clonidine, and placebo [14]. Our study's control group EA rate (50%) is consistent with literature reporting 22-55% incidence in nasal procedures, validating our patient cohort's risk profile and the clinical significance of our intervention. The α_2_-adrenergic selectivity of dexmedetomidine modulates arousal-regulating noradrenergic pathways in the brainstem and limbic regions, directly opposing the hyperactivity characteristic of sevoflurane-induced emergence delirium [15]. Additionally dexmedetomidine's anti-inflammatory effects through reduced cytokine dysregulation preserve neural homeostasis during volatile anesthetic emergence. Our sedation quality analysis using the Ramsay Scale demonstrated superior control in the dexmedetomidine group (64.4% Grade 2 vs. 28.9%, p = 0.000) reflecting the distinction between hypnotic-induced sedation and dexmedetomidine's "cooperative sedation" phenotype clinically advantageous for maxillofacial patients requiring early mobilization and subtle neurological assessment. Our findings show comparable postoperative pain, nausea-vomiting, and cough scores between groups, contrasting with agents like propofol that prolong sedation or induce over-recovery, which delays discharge and increases morbidity. Recent evidence from nasal surgery (anatomically similar to maxillofacial fields) using alternative approaches including remimazolam-based total intravenous anesthesia (TIVA) achieving 0% EA versus 12.2% with sevoflurane underscores that while pharmacological alternatives exist, dexmedetomidine remains superior in averting EA without prolonging PACU discharge [16]. Multimodal interventions combining dexmedetomidine (0.4 µg/kg/h infusion) with ropivacaine topical anesthesia and remifentanil achieved superior cough suppression while maintaining comparable hemodynamic stability and adverse event profiles [17]. A critical safety consideration emerged: bradycardia occurred significantly more frequently in the dexmedetomidine group (22.2% vs. 6.7%, p = 0.036). Recent meta-analyses report a 2.38-fold increased odds of bradycardia (95% CI 1.77-3.21) with dexmedetomidine, though the FDA Adverse Event Reporting System analysis reveals bradycardia as the most common reported adverse event yet clinically benign at conventional dosing (0.2-0.7 µg/kg/h), with severe complications (cardiac arrest, syncope) remaining rare [18]. In cardiac surgery populations, dexmedetomidine-induced bradycardia increased risk 3.4-fold but did not translate to increased mortality or atrial fibrillation [19]. In our ASA I-II maxillofacial cohort without preoperative cardiac disease, observed bradycardia was manageable without intervention, affirming clinical acceptability of this pharmacokinetic effect. Hypotension while slightly elevated (17.8% vs. 6.7%), did not achieve statistical significance (p = 0.108), indicating dexmedetomidine's superior hemodynamic tolerance compared to propofol, which causes profound vasodilation requiring vasopressor support in up to 39% of recipients. Supporting this a pediatric RCT in cleft surgery reported much less agitation with dexmedetomidine versus sevoflurane, no extubation delay and only a small increase in time to full consciousness [20]. Intraoperative hemodynamic monitoring revealed preserved oxygen saturation and overall stability despite lower intraoperative arterial pressures and heart rate a "sympatholytic stability" distinguishing α_2_-agonists from other sedatives. The modest dexmedetomidine-induced hemodynamic depression is physiologically benign and well-compensated in the perioperative setting, particularly in non-cardiac ASA I-II populations. Our study's cross-sectional observational design and ASA I-II homogeneity limit generalizability to higher-risk cohorts; however, consistent findings across diverse Asian and international surgical populations strengthen the validity of our conclusions for maxillofacial surgical practice. Dexmedetomidine at 0.4 µg/kg/h represents a safe, effective adjuvant for preventing emergence agitation in adult maxillofacial surgery under sevoflurane anesthesia, with bradycardia representing a predictable, manageable adverse effect outweighed by substantial benefits in recovery quality and postoperative comfort.

## Conclusion:

Intraoperative dexmedetomidine significantly reduces emergence agitation and improves sedation quality in adult maxillofacial surgeries. Its infusion protocol (1 µg/kg loading, 0.4 µg/kg/hr maintenance) was effective without increasing pain, nausea or vomiting. Hemodynamic stability was better in the Dexmedetomidine group, though bradycardia was more frequent and needs monitoring. Thus, it becomes a useful and well-tolerated adjuvant for smoother emergence from general anaesthesia.
